# The Use of Electronic Nose in the Quality Evaluation and Adulteration Identification of Beijing-You Chicken

**DOI:** 10.3390/foods11060782

**Published:** 2022-03-08

**Authors:** Jingru Chen, Wenjie Yan, Yu Fu, Liang Wang, Xueze Lv, Ruitong Dai, Xingmin Li, Fei Jia

**Affiliations:** 1 College of Food Science and Nutritional Engineering, China Agricultural University, No. 17 Qinghua East Road, Haidian District, Beijing 100083, China; 18800120616@163.com (J.C.); dairuitong@vip.sina.com (R.D.); 2 College of Biochemical Engineering, Beijing Union University, Beijing 100023, China; meyanwenjie@buu.edu.cn; 3 Department of Food Science, Southwest University, Chongqing 400715, China; fuy987@swu.edu.cn; 4 Animal Husbandry Station of Beijing, No. 17 Beiyuan Road, Chaoyang District, Beijing 100101, China; carspstp@126.com (L.W.); lvxueze0310@163.com (X.L.); 5 Department of Biological and Agricultural Engineering, University of Arkansas, Fayetteville, AR 72701, USA

**Keywords:** Beijing-you chicken, meat quality, flavor, GC-MS, electronic nose

## Abstract

The objective of this study was to reveal the secrets of the unique meat characteristics of Beijing-you chicken (BJY) and to compare the difference of quality and flavor with Luhua chicken (LH) and Arbor Acres broiler (AA) at their typical market ages. The results showed the meat of BJY was richer in essential amino acids, arachidonic acid contents, inosine monophosphate (IMP), and guanosine monophosphate (GMP). The total fatty acid and unsaturated fatty acid contents of BJY chicken and LH chicken were lower than that of AA broilers, whereas the ratios of unsaturated fatty acids/saturated fatty acids (2.31) and polyunsaturated fatty acids/monounsaturated fatty acids (1.52) of BJY chicken were the highest. The electronic nose and SPME-GC/MS analysis confirmed the significant differences among these three chickens, and the variety and relative content of aldehydes might contribute to a richer flavor of BJY chicken. The meat characteristics of BJY were fully investigated and showed that BJY chicken might be favored among these three chicken breeds with the best flavor properties and the highest nutritional value. This study also provides an alternative way to identify BJY chicken from other chickens.

## 1. Introduction

Beijing-you (BJY) chicken, as one of 27 rare breeds in China, is increasingly favored by Chinese customers for its superior meat and egg qualities [1]. In 2020, BJY chicken was awarded the “Agro-product Geographical Indications” by the Ministry of Agriculture of the P.R.C., due to its distinctive appearance, strong viability, and stable genetic performance [2]. As a result, the price of BJY chicken of approximately RMB 70/500 g is much higher than other commercially available chickens. However, very few researchers focus on investigating the quality and flavor characteristics of BJY chicken and adulteration identification technology to differentiate BJY chicken from other low-value chickens, which could help BJY chicken to remain competitive in the market.

Consumer acceptance of chicken meat relies on its quality, such as visual appearance, smell, tenderness, and juiciness [3]. Nowadays, nutrition and sensory quality are noted as key factors in the consumer perception of chicken meat [4,5]. Among them, the flavor, including taste and odor, is one of the most important characteristics [6]. Taste is the sensation that the tongue receives when it contacts with soluble substances, such as free amino acids and nucleotides, while the odor is sensed through the olfactory organs [7]. Gas chromatography-mass spectroscopy (GC/MS) is the most commonly technique to analyze odor profiles of meat products, which could provide an accurate approach for the qualitative and quantitative analysis of volatile compounds [8]. Solid-phase microextraction (SPME) has the advantages of simplicity of operation, speed, a solvent-free nature, analyte separation, and preconcentration [6]. The electronic nose is a device that consists of a multi-sensor array and multidimensional signal processing of the array signal by pattern recognition algorithms [9] and can measure the presence of volatile compounds related to the meat aroma. In this technique, the analytic process does not concentrate on the identification and quantification of the volatile compounds but rather on the quantitative description of the complete aroma profile, including the relationships between its components [10]. Compared with traditional sensory evaluation and physicochemical techniques, which are expensive and time consuming, the electronic nose provides an efficient, rapid, non-destructive, and real-time testing, and this technique is widely used in freshness evaluation [11], shelf-life investigation [12], meat product authenticity [13,14], and flavor distinction [15,16].

Compared with the commercial fast-growing broiler breeds, such as Arbor Acres broiler (AA), the slow-growing native chicken is more preferred in Asian areas because of its high nutrition value, unique organoleptic characteristics, and special flavors [17,18,19]. An increasing number of people prefer to buy native chickens, even when the price is approximately two to four times higher than the commercial broiler. Luhua (LH) chicken is a Chinese local chicken breed that is widely raised in rural areas in China. Generally, local chickens in China, including BJY chicken and LH chicken, will be used for meat production at 30–40 weeks of age [1]. The imported commercial broiler, however, usually takes approximately 42 days in growth, which makes it the main chicken meat in Chinese supermarkets, especially in the fast-food industry.

In this study, the quality and flavor of BJY chicken were fully investigated, and commercial broilers (AA) and common and widely raised Chinese chicken breed chicken (LH) were selected as two typical chickens to investigate the differences in meat quality and flavor. Specifically, both nonvolatile compounds (fatty acids, amino acids, and IMP and their relative contents) and volatile organic compounds (VOCs) of the three chickens, which contribute to the sensory properties, were measured and compared in detail. Meanwhile, the electronic nose was also used to characterize and classify the differences of volatile compounds of these three chicken breeds. By comparing the difference in flavor and quality of these three types of chicken, we attempt to reveal the meat characteristics of BJY chicken and provide a possible identification method.

## 2. Materials and Methods

### 2.1. Sample Collection

BJY chicken (aged 240 days with a live weight of 2.0–2.5 kg) and LH chicken (aged 240 days with a live weight of 2.0–2.5 kg) were purchased from Beijing Xiqing Minfeng Agricultural Development Co., Ltd. (Beijing, China). Experimental animals of these two breeds (5 birds per breed, respectively) were slaughtered by conventional neck cut, bled for 2 min, defatted, and eviscerated. The breast (pectoralis major) muscles were dissected carefully. All operations were carried out in accordance with the Guidelines for experimental Animals established by the Ministry of Science and Technology (Beijing, China). Five AA broilers (aged 42 days and with live weight 1.5–2.0 kg) were purchased from Beijing Huadu Poultry Breeding Co., Ltd. (Beijing, China).

### 2.2. Determination of Intramuscular Fat (IMF) and Crude Protein (CP)

IMF content was measured as described by Ju et al. [20], with slight changes. A minced meat sample (5 g) of each chicken was mixed with 50 mL of petroleum ether to ultrasonically extract the IMF for 45 min. Extracted IMF was filtered, dried with anhydrous Na_2_SO_4_, and concentrated by rotary evaporator in a 70 °C water bath. The above steps were repeated three times to obtain IMF. The results were expressed as the weight percentage of wet muscle tissue.

The nitrogen (N) content was assayed using the Kjeldahl method, which was used to calculate CP by multiplying N × 6.25. The results were expressed as the weight percentage of wet muscle tissue.

### 2.3. Determination of Nucleotide Compound Contents

Nucleotide content was estimated as described by Jung et al. [21], with slight changes. A minced meat sample (5 g) of each chicken was mixed with 20 mL of 5% (volume/volume) perchloric acid to extract nucleic acids. Extracted nucleic acids were centrifuged at 9200× *g* for 10 min. The supernatant was then adjusted to pH 6.4 with 1 mol/L KOH. The supernatant was placed in a volumetric flask and adjusted to a volume of 25 mL with distilled water, filtered through a 0.22 μm membrane filter, and analyzed for adenosine triphosphate (ATP), and its related compounds were measured by HPLC (Shimadzu, Kyotos, Japan) equipped with an SPD-10A (V) detector, VP-CDS C18 column (4.6 mm id × 250 mm, 5 μm). The sample (10 μL) was injected at a flow rate of 0.7 mL/min, and the peak was detected at 254 nm. The amounts of ATP, adenosine diphosphate (ADP), adenosine monophosphate (AMP), IMP, inosine (HxR), hypoxanthine (Hx), and GMP were determined and calculated based on the standard ATP, ADP, AMP, IMP, HxR, Hx, and GMP. All standards reagents were purchased from Sigma (Merck, Darmstadt, Germany). The results were expressed as milligram of nucleotides contents per 100 g of wet muscle tissue.

### 2.4. Determination of Amino Acid Contents

Amino acid content was estimated based on the previous methods reported by Li et al. [22]. Breast muscle samples (2 g) were freeze-dried and ground for extraction, then amino acids were determined in triplicate by an Amino Acid Analyzer (Sykam, Munich, Germany). The results were expressed as milligrams of amino acids per 100 g of wet muscle tissue.

### 2.5. Determination of Fatty Acids

Fatty acid content was determined by gas chromatography, as reported by Gecgel [23]. Breast muscle samples were freeze dried and ground and then analyzed using an HP6890 gas chromatography system (Hewlett-Packard, Palo Alto, CA, USA). The results were expressed as milligrams of fatty acids per 100 g of wet muscle tissue. 

### 2.6. Determination of VOCs

The VOCs were determined by an automated injector using the method introduced by Li et al. [24], with some modifications. Meat samples weighing 1 g were placed into 20 mL headspace vials prior to being pre-heated at 60 °C for 20 min for system equilibration. PDMS/DVB fiber (65 mm) was inserted and exposed to the headspace of the vial. After 30 min, the fiber was withdrawn and inserted into the injection port of a GC (Shimadzu, Kyoto, Japan) injector at 200 °C for 2 min for desorption. 

In a GC-MS system equipped with an MS detector (Shimadzu, Kyoto, Japan), VOCs were separated by a capillary DBWAX column (30 m × 0.25 mm × 0.25 mm). The temperature of the GC oven was first kept at 40 °C for 3 min, increased 5 °C/min to 120 °C, and then increased 10 °C/min to 200 °C and held for 13 min. The injections were performed in splitless mode, and the carrier gas was helium with a flow rate of 1 mL/min. The collection of MS data was acquired at a full scan range from 35 to 500 m/z. The transfer lines and MS source remained at 250 °C and 200 °C, respectively. The VOCs were identified by matching mass spectra or retention time with those in the National Institute of Standards and Technology (NIST) 11 spectral database and were quantified by the area normalization method.

### 2.7. Electronic Nose Evaluation

**Figure 1:**
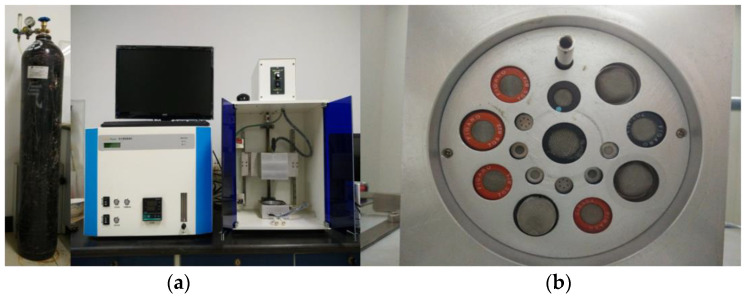
Electronic nose device (**a**) and gas sensor distribution (**b**).

The volatile compounds of chicken breast meat were analyzed using an E-Nose 10001 system (developed by the College of Information and Electrical Engineering, China Agricultural University). The E-Nose 10001 electronic nose system mainly consists of the following parts: data acquisition part, data conditioning part, interface circuit part, and computer host. The hardware part includes a gas sensor array, a signal conditioning circuit board, an A/D conversion interface, and a computer, as shown in Figure 1. Previous studies have proven that, after the sensor array optimization and feature optimization, E-Nose 10001 can distinguished pork from different manufacturers, and also the parts and storage conditions. Compared with the PEN3 electronic nose of Airsense, the results of E-Nose 10001 are more accurate.

The electronic nose was equipped with 16 different metal oxide sensors: TGS824, TGS822, TGS825, TGS880, TGS812, TGS831, TGS813, TGS830, TGS822TF, TGS2600, TGS2620, TGS2611, TGS2602, TGS2620, TGS2610, TGS2201. Before the measurements were taken, the headspace gases were injected at a flux speed of 3 L/min for 60 s. Then, 5 g of minced chicken breast samples (3 birds per breed, respectively) were placed in a vial at a temperature of 40 °C. The gases in the headspace of the sample were pumped into a gas sensor chamber at the same speed. The electronic nose measurement interval was 0.05 s. Electronic nose real-time responses to chicken breast samples were recorded with 5 replicates. 

### 2.8. Reagent Section

All reagents and solvents used are listed in Table 1.

### 2.9. Statistical Analysis

Mean and standard deviations were calculated and subjected to analysis of variance. Duncan’s test was used to test for differences between means, and the significance was defined at *p* < 0.05 using SPSS 18.0 software (Chicago, IL, USA). The discriminant results of the electronic nose sensors for different chicken breast meat were based on canonical discriminant analysis (CDA).

## 3. Results and Discussion

### 3.1. Contents of Muscle IMF and CP

**Figure 2:**
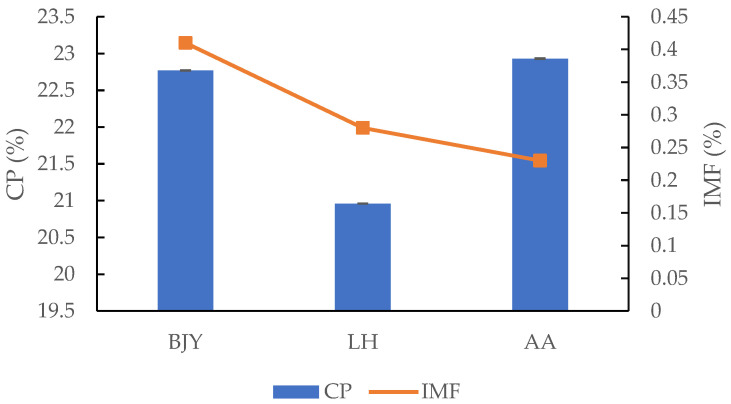
IMF and CP contents of different breeds of chicken breast.

The IMF and CP contents of different breeds of chicken breast are presented in Figure 2. The CP contents of BJY and AA broilers were slightly higher than that of LH chicken, and there were no significant differences between BJY and AA broilers on the CP contents. Comparison of breeds revealed that breast IMF content was higher (*p* < 0.05) in BJY chicken (0.41%), whereas IMF content was intermediate in LH chicken (0.28%) and lowest in the AA broilers (0.23%). IMF content has a close relationship with good flavor, juiciness, and improved tenderness of meat [25]. These similar results were also found in previous studies, where native chicken breeds had higher IMF contents than imported commercial broilers [26,27]. Both genes and environment could influence the IMF content of meat [28]. Ranran et al. [29] revealed the embryonic development-related proteome and metabolome signatures in the breast muscle and intramuscular fat of fast-growing (BJY) and slow-growing chickens. 

### 3.2. Contents of Nucleotide Compound

From Table 2, the IMP contents of the breast meat from BJY and LH, which are 459.77 mg/100 g and 413.49 mg/100 g, respectively, were significantly higher than AA broilers (247.25 mg/100 g). Some other studies have shown similar results; for example, Jung et al. [20] and Tang et al. [30] found that slow-growing chicken breeds in Korea and China had higher contents of IMP than fast-growing commercial broilers. The differences in IMP content among different breeds may be explained by the effects of genotype, age, or their interaction. In addition, there was genetic effect on IMP content in chicken meat among indigenous breeds [31]. Li et al. [22] found that the content of IMP from Wenchang chicken, another indigenous chicken from China, was highly related to their genotype. It has been widely accepted that IMP is the most important nucleotide-based flavor precursor and can produce a synergistic effect conjugated with monosodium glutamate [32].

GMP is another important nucleotides that provides pleasant flavor for meat and can also be used as a flavor enhancer [33]. The content of GMP in BJY (5.87 mg/100 g) and LH (6.15 mg/100 g) was significantly higher than that in AA (4.19 mg/100 g). The differences of GMP content may be explained by the effects of genotype, feed, age and feeding condition [34,35,36,37,38]. Over time after slaughter, IMP can degrade to HxR and Hx. It was reported that the accumulation of HxR and Hx led to a decrease in freshness [39]. Therefore, the relatively high IMP and GMP contents and lower content of Hx and HxR in the BJY breast meat may produce a better flavor compared with LH chicken and AA broilers.

### 3.3. Contents of Amino Acids

Free amino acids are of great importance in eating quality due to their specific tastes and important flavor and flavor precursor substance in chicken meat [40]. The amino acid profiles of breast meat from BJY, LH, and AA broilers are depicted in Table 3.

It is clear that the predominant amino acids in the essential fraction were leucine and lysine in all chicken breeds. In the nonessential fraction, glutamic acid was the richest amino acid. Similar results were also reported in previous studies. Chen et al. [5] found that glutamic acid in the nonessential fraction and lysine and leucine in the essential fraction were also major amino acids in 817 crossed chickens (a commercial Chinese crossed chicken), AA broilers, and Hyline Brown (commercial spent hens). The same results were also found in some other meat such as eland, cattle [41], and goose [42].

In this study, different chickens were significantly different (*p* < 0.05) in their amino acid contents, with the exception of glutamic acid, glycine, alanine, valine, leucine, arginine, and proline in the breast. Regarding total essential amino acids, significant differences were found among all three groups. AA broilers contained relatively higher total essential amino acids (315.20 mg/100 g), followed by BJY and LH (296.02 mg/100 g and 263.33 mg/100 g, respectively). However, the essential amino acid contents of BJY chicken showed significantly higher values than LH and AA broilers in breast meat. Glutamic acid is an important flavor compounds of meat, which is an important contributor to the fresh taste of meat [43]. BJY chicken had the highest content of glutamic acid (82.02 mg/100 g) among these three chicken breeds. The content of glutamic acid in LH chicken was slightly higher than that in AA broilers, but there was no significant difference. Wattanachant et al. [44] also confirmed that the breast meat of Thai native chickens had higher glutamic acid compared with broiler chickens. Therefore, the results indicate that the chicken breed considerably affects the amino acid composition considerably. The high content of essential amino acids in the breast muscles of BJY chicken might suggest that the BJY chicken has more nutritional value to humans than LH and AA chicken.

### 3.4. Contents of Fatty Acids

The fatty acid composition of meat was affected by many factors, such as age and genotype [21,29]. Furthermore, dietary manipulation can alter the fatty composition and fatty acid contents [45]. The different fatty acid compositions of muscles most likely affect lipid stability and flavor. Table 4 summarizes the fatty acid profiles in the breast muscle of these three breeds. The major components measured in the chicken meat from BJY, LH, and AA were linoleic acid (C 18:2), oleic acid (C 18:1), and palmitic acid (C 16:0), which accounted for approximately 70% of total fatty acids; this is consistent with the results reported by previous studies [5]. Regarding the total saturated acids (SFA), LH chickens showed significantly lower values (32.21 mg/100 g) in comparison to BJY and AA broilers, while there were no significant differences between BJY and AA broilers. However, the contents of unsaturated fatty acids (UFA) in AA broilers were significantly higher than those in BJY and LH chickens. Regarding UFA/SFA and PUFA/monounsaturated fatty acids (MUFA), the ratios were both significantly higher in BJY chickens than in LH chickens and AA broilers, which suggests that the composition of fatty acids in BJY chickens breast meat were better than that of same-age LH chickens and fast-growing AA broilers.

The arachidonic acid content (C 20:4) in BJY chickens was more than twice than that of AA broilers, which were 14.55 mg/100 g and 6.25 mg/100 g, respectively. It was 10.87 mg/100 g C 20:4 in LH chickens, which was higher than that in AA broilers but lower than that in BJY chickens. Arachidonic acid can directly participate in intracellular signaling transduction and affect other signaling pathways to control cellular biological activity, which is a very important intracellular second messenger [46]. In addition, it was reported that, when the arachidonic acid composition was increased by supplementation with an acid enriched oil diet, the flavor intensity, total taste intensity, umami, and aftertaste of broiler muscle also increased [47]. Jeon et al. [48] found that the breast meat of Korean indigenous chickens had higher arachidonic acid contents than that of broilers. Zhao et al. [3] reported that the breast meat from BJY chickens contained significantly higher amounts of arachidonic acid than that of commercial fast-growing AA broilers at their market age. These results suggest that BJY chickens have better flavor properties and more nutritional value to humans than that of LH chickens and AA broilers.

### 3.5. VOCs Analysis

Table 5 shows the comparison of VOCs relevant contents of different types of chicken breast; 20, 25, and 19 VOCs were detected in the BJY, AA and LH, respectively. VOCs chromatograms of BJY, LH, and AA were showed in Figures S1–S3, respectively. There were no complicated heterocyclic compounds such as pyrroles and pyrazines in this study, which might be related to the lower maturation temperature (60 °C). Among the VOCs, 5 compounds were detected in all three types of chicken, including1, 3-bis (1,1-dimethylethyl)-benzene, heneicosane, tetradecane, 2,4-bis (1,1-dimethylethyl)-enol, and hexadecanal. The volatile flavor substances together affect the final sensory quality of chicken meat. Jiang [49] found that the volatile compounds in Avain broilers, fast-da Yellow chicken, and BJY also contained tetradecane and palmaldehyde. It was predicted that tetradecane and hexadecanal were common volatile substances in chicken meat.

Table 6 shows the quantity and relative content of VOCs in different types of chicken meat. Hydrocarbons, alcohols, and aldehydes were major VOCs in all three chicken samples, and their contribution to chicken flavor varies with the substance threshold [50]. Hydrocarbons were mainly derived from the cleavage of fatty acid alkoxy radicals, and the differences in the contents might be caused by the differences in their precursor fatty acids. The relative contents and types of hydrocarbon compounds in the three muscles were quite different, which were consistent with the previous results of the fatty acid contents. The aroma threshold of hydrocarbons is relatively high, and it is generally believed that they have little direct contribution to the flavor of meat [51]. Alcohols are mainly derived from the oxidation and degradation of lipids, which have pleasant fruity and floral odors [52]. Among them, 1-octen-3-ol has a mushroom-like smell and is the product of arachidonic acid oxidation by lipoxygenase [24], which was detected in both BJY and LH. In this study, the quantity and relative contents of alcohols in BJY and LH chicken were higher than AA chicken. Aldehydes are aliphatic compounds produced by lipid oxidization and thermal degradation [53], which are usually considered to be the major flavor contributors to meat products due to their low thresholds [54]. Previous studies have proved that aldehydes such as nonanal and decanal were characteristic aroma substances of chicken [55]. Nonanal was mainly formed by the oxidation of linoleic acid [56]. The fat content of BJY was relatively higher, so the variety and relative content of the aldehydes detected were also higher than LH and AA, contributing to a richer flavor of the chicken. In addition, saturated linear aldehydes with high molecular weight may produce pungent odors. The content of Pentadecanal and Hexadecanal in AA was relatively high. Li et al. have found that Tetradecanal might be one of the sources of the unpleasant earthy smell in fish [24]. 

### 3.6. Electronic Nose Analysis

**Figure 3:**
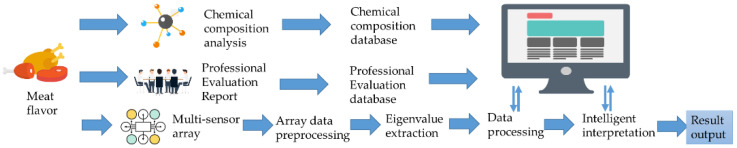
Simulation of the olfaction by electronic nose technology.

The increase in meat production and the need for rapid detection have contributed to the development of simple, fast, accurate, and inexpensive methods to evaluate the classification or quality of meat [48]. Figure 3 shows how the electronic nose simulates the olfaction.

The multivariate recognition algorithm to process the multi-sensor array signals is based on the linear discriminant analysis method [57]. Because the process is simple and economical, sample preparation is minimal, and reading and interpretation of the measurements are clear, the electronic nose has become a viable alternative to conventional analysis [11,58] and has been applied to evaluate the shelf-life of livestock products [59], and to analyze meat quality [60].

**Figure 4:**
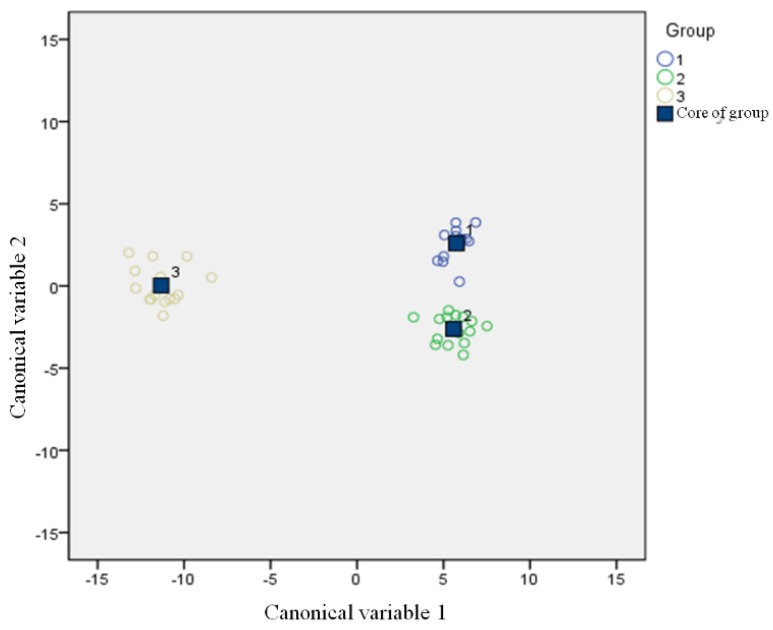
Canonical discriminant analysis of the three types of chicken breast: Group 1, 2, and 3 represent BJY, LH, and AA, respectively.

In the present study, there were nine eigenvalues of 16 sensors of the electronic nose, including means, integral value, differential value, range, quadratic coefficient, primary coefficient, halfwidth, primary coefficient of logarithmic regression function, and constant term of logarithmic regression function, which were used to analyze these three breeds of chicken [61]. They could be accurately identified with the CDA to analyze the acquired data in order to evaluate the overall flavor characteristics among the three chicken breeds. As shown in Figure 4, the CDA based on the 144 parameters derived the first and second canonical variables (CN1 and CN2, respectively). CAN1 explained 93.4% of the variability and was able to differentiate BJY and LH chickens from AA broilers. Furthermore, CAN2 was able to separate BJY from LH chickens. The results indicate that the distance between the core of BJY chickens and LH chickens was relatively close, but it could also be completely separated. The possible reason is that these two breeds of chicken were fed in the same environment and at the same age.

The above studies have shown that electronic nose technology can be used to successfully distinguish the volatile compounds among these three breeds of chickens. This method exhibits satisfactory results using appropriate pattern recognition techniques for data analysis, which provides a gratifying analytical method for Beijing-you chicken identification. With the advantages of high sensitivity and excellent selectivity, the electronic nose has broad application prospects for the detection of meat adulteration in the future.

## 4. Conclusions

This study demonstrated several differences among BJY chickens, LH chickens, and AA broilers in terms of nutritional and sensory properties. At their typical market ages, 240-day-old BJY was preferable to 240-day-old LH and 42-day-old AA broilers due to its higher protein content, higher IMP and GMP content, and lower Hx and HxR content. Noticeably, BJY chicken showed an especially high arachidonic acid (14.55 mg/100 g) and essential amino acids content (127.84 mg/100 g). These characteristics might contribute to better flavor properties and higher nutritional value to humans, which would meet the preference of those consumers in the current market. The relative contents and varieties of VOCs, detected by SPME-GC/MS, were quite different between the three chicken breeds, which resulted in differences in the flavors. The variety and relative content of aldehydes might contribute to a richer flavor of the chicken. Furthermore, the electronic nose results also confirmed that there were significant differences between the breast meat of these three breeds of chicken by canonical discriminant analysis. The results revealed the meat characteristics of BJY chicken and perhaps provide a possible adulteration identification method, which can help consumers choose premium chicken meat in the market.

## Figures and Tables

**Table 1:** List of reagents and solvents used.

Reagents	Types	Manufacturer
ATP	Chromatographically pure	Sigma
ADP	Chromatographically pure	Sigma
AMP	Chromatographically pure	Sigma
IMP	Chromatographically pure	Sigma
GMP	Chromatographically pure	Sigma
HxR	Chromatographically pure	Sigma
Hx	Chromatographically pure	Sigma
Methanol	Chromatographically pure	Fisher Scientific
N-hexane	Chromatographically pure	Fisher Scientific
N-heptane	Chromatographically pure	Fisher Scientific
Ethanol	Analytically pure	Solarbio
Sulfosalicylic acid	Analytically pure	Solarbio
Potassium hydroxide	Analytically pure	Solarbio
Phthalaldehyde	Analytically pure	Solarbio
Perchloric acid	Analytically pure	Solarbio
Potassium dihydrogen phosphate	Analytically pure	Solarbio
Dipotassium phosphate	Analytically pure	Solarbio
Petroleum ether	Analytically pure	Solarbio
Anhydrous sodium sulfate	Analytically pure	Solarbio

**Table 2:** Nucleotide contents of different types of chicken breast (mg/100 g).

Nucleotides	BJY	AA	LH
GMP	5.87 ± 0.501 ^a^	4.19 ± 0.01 ^b^	6.15 ± 0.37 ^a^
IMP	459.77 ± 24.98 ^a^	247.25 ± 20.22 ^b^	413.49 ± 25.42 ^a^
Hx	8.63 ± 0.35 ^a^	63.46 ± 4.47 ^c^	13.81 ± 0.50 ^b^
HxR	62.27 ± 3.15 ^a^	147.26 ± 9.74 ^b^	60.73 ± 4.04 ^a^

^a,b,c^ Means within a row with different superscripts differ significantly (*p* < 0.05).

**Table 3:** Free amino acid contents of different types of chicken breast (mg/100 g).

Amino Acid (mg/100 g)	BJY	LH	AA
Asp (Aspartic acid)	7.04 ± 0.28 ^b^	1.13 ± 0.09 ^a^	18.81 ± 0.19 ^c^
Thr (Threonine)	7.71 ± 0.22 ^b^	6.45 ± 0.18 ^a^	10.34 ± 0.26 ^c^
Ser (Serine)	4.56 ± 0.04 ^a^	6.16 ± 0.17 ^b^	7.84 ± 0.40 ^c^
Glu (Glutamic acid)	82.02 ± 4.31 ^a^	64.66 ± 3.37 ^b^	57.95 ± 0.57 ^b^
Gly (Glycine)	10.97 ± 0.49 ^a^	8.93 ± 0.00 ^b^	11.38 ± 0.21 ^a^
Ala (Alanine)	15.82 ± 0.71 ^a^	14.56 ± 0.47 ^a^	21.95 ± 0.50 ^b^
Cys (Cystine)	0.76 ± 0.04 ^a, b^	0.92 ± 0.06 ^b^	0.71 ± 0.06 ^a^
Val (Valine)	19.12 ± 0.81 ^a^	18.98 ± 0.95 ^a^	14.98 ± 0.93 ^b^
Met (Methionine)	9.05 ± 0.37 ^b^	6.14 ± 0.26 ^a^	11.88 ± 0.14 ^c^
Ile (Isoleucine)	21.10 ± 0.62 ^a^	18.92 ± 0.55 ^b^	16.19 ± 0.16 ^c^
Leu (Leucine)	30.10 ± 0.60 ^a^	26.44 ± 0.18 ^b^	25.74 ± 0.55 ^b^
Tyr (Tyrosine)	19.00 ± 0.90 ^a, b^	18.33 ± 0.01 ^a^	20.21 ± 0.23 ^b^
Phe (Phenylalanine)	19.60 ± 0.05 ^a^	18.22 ± 0.48 ^b^	14.43 ± 0.14 ^c^
His (Histidine)	11.14 ± 0.25 ^a^	15.17 ± 0.27 ^b^	18.30 ± 0.14 ^c^
Lys (Lysine)	21.18 ± 0.26 ^a^	19.19 ± 0.89 ^b^	20.10 ± 0.31 ^ab^
Arg (Arginine)	8.96 ± 0.57 ^a^	10.00 ± 0.46 ^a^	19.79 ± 0.12 ^b^
Pro (Proline)	7.94 ± 1.0 ^a^	9.16 ± 0.29 ^a^	24.64 ± 0.05 ^b^
EAA	127.84 ± 0.04 ^a^	114.34 ± 2.76 ^b^	113.64 ± 0.58 ^b^
Total	296.02 ± 3.70 ^b^	263.33 ± 0.25 ^a^	315.20 ± 1.77 ^c^

^a,b,c^ Means within a row with different superscripts differ significantly (*p* < 0.05). EAA = essential amino acids (including threonine, valine, methionine, isoleucine, leucine, phenylalanine, and lysine).

**Table 4:** Fatty acid contents of different types of chicken breast (mg/100 g).

Fatty Acid (mg/100 g)	BJY	LH	AA
Palmitic Acid C 16:0	19.02 ± 1.12 ^a^	21.88 ± 2.41 ^a^	65.75 ± 2.83 ^b^
Stearic Acid C 18:0	16.37 ± 1.87 ^a^	10.34 ± 2.25 ^a^	27.00 ± 2.12 ^b^
Oleic Acid C 18:1	26.90 ± 3.37 ^a^	15.9 ± 1.12 ^a^	91.25 ± 5.30 ^b^
Linoleic Acid C 18:2	34.60 ± 3.62 ^b^	15.37 ± 7.12 ^a^	76.50 ± 4.95 ^c^
Arachidonic Acid C 20:4	14.55 ± 0.71 ^a^	10.87 ± 0.75 ^b^	6.25 ± 0.35 ^c^
Nervonic Acid C 24:1	5.43 ± 1.31 ^a^	6.89 ± 1.12 ^a^	3.63 ± 1.94 ^a^
Total	116.86 ± 7.97 ^b^	79.24 ± 3.37 ^a^	270.38 ± 17.50 ^c^
SFA	92.75 ± 4.95 ^a^	32.21 ± 4.66 ^b^	92.75 ± 4.95 ^a^
MUFA	32.33 ± 2.06 ^a^	22.79 ± 2.25 ^a^	94.88 ± 7.25 ^b^
PUFA	49.15 ± 2.91 ^b^	26.24 ± 6.37 ^a^	82.75 ± 5.30 ^c^
UFA	81.48 ± 4.97 ^b^	49.03 ± 4.12 ^a^	177.63 ± 12.55 ^c^
PUFA/MUFA	1.52 ± 0.01 ^a^	1.17 ± 0.40 ^a^	0.87 ± 0.02 ^a^
UFA/SFA	2.31 ± 0.05 ^b^	1.55 ± 0.35 ^a^	1.92 ± 0.03 ^c^

^a,b,c^ Means within a row with different superscripts differ significantly (*p* < 0.05). SFA = saturated fatty acids; MUFA = monounsaturated fatty acids; PUFA = polyunsaturated fatty acids; UFA = unsaturated fatty acids.

**Table 5:** Comparison of VOCs relevant contents of different types of chicken breast (%).

VOCs	BJY	LH	AA
**Hydrocarbons**			
(1-Hexadecylheptadecyl)-Cyclohexane	2.13 ± 0.01	—	—
1,3-Bis(1,1-dimethylethyl)-Benzene	12.72 ± 0.05	17.68 ± 0.00	0.70 ± 0.01
2-Methylhexacosane	—	—	2.70 ± 0.30
(2,3-Dimethyldecyl)-Benzene	2.27 ± 0.01	—	—
2,4-dimethyl-1-Decene	—	3.82 ± 0.00	—
2,4-Dimethyl-Eicosane,	—	—	0.39 ± 0.01
2,6,10,14-Tetramethyl-Pentadecane	—	—	11.19 ± 0.03
2-Methyl-Dodecane	—	2.45 ± 0.01	—
2-Methyl-Hexadecane	—	—	6.74 ± 0.13
2-Methyltetracosane	2.53 ± 0.02	2.72 ± 0.16	—
3-Methyl-Heptadecane	—	—	5.06 ± 0.51
8-Heptyl-Pentadecane	—	—	2.63 ± 0.18
8-methyl-1-Undecene	—	2.37 ± 0.00	—
8-Methyl-Heptadecane	—	—	1.60 ± 0.04
Decyl-Cyclohexane	—	—	3.85 ± 0.04
Decyl-Cyclopentane	—	—	1.18 ± 0.01
Undecyl-Cyclohexane	—	—	2.19 ± 0.03
Dodecylcyclohexane	—	—	2.03 ± 0.11
Eicosane	—	0.74 ± 0.00	7.59 ± 4.28
Heneicosane	13.36 ± 0.66	6.32 ± 0.40	19.05 ± 0.59
Heptadecane	—	—	6.66 ± 0.36
Hentriacontane	—	5.60 ± 0.12	—
Hexadecane	—	—	4.90 ± 0.99
Octadecane	—	—	3.73 ± 0.08
Squalane	—	—	6.00 ± 0.88
Tetradecane	1.28 ± 0.01	3.99 ± 0.01	0.82 ± 0.01
**Alcohols**			
1-Dodecanol	2.74 ± 0.00	4.18 ± 0.00	—
1-Octen-3-ol	9.46 ± 0.04	14.62 ± 0.35	—
2-(2-Ethoxyethoxy)-Ethanol	13.76 ± 2.33	6.93 ± 0.02	—
2-(Hexadecyloxy)-Ethanol	2.74 ± 0.00	—	—
2-methyl-1-Decanol	—	2.30 ± 0.07	—
2-Methyl-1-Hexadecanol	2.67 ± 0.12	—	—
5-Methyl-2-(1-methylethyl)-1-Hexanol	—	4.28 ± 0.07	—
Octahydro-4a(2H)-Naphthalenemethanol	—	—	0.41 ± 0.01
**Phenols**			
2,4-Bis(1,1-dimethylethyl)-enol	8.92 ± 0.08	13.36 ± 0.03	0.06 ± 0.01
**Aldehydes**			
Dodecanal	2.28 ± 0.01	1.38 ± 0.06	—
Hexadecanal	0.17 ± 0.01	2.31 ± 0.05	2.45 ± 0.10
Nonanal	—	1.84 ± 0.01	—
Pentadecanal	0.12 ± 0.00	—	2.92 ± 0.01
Tridecanal	7.64 ± 0.10	—	—
Tetradecanal	2.04 ± 0.01	—	—
**Esters**			
[1,1′-Bicyclopropyl]-2-octanoic acid, 2′-hexyl-, methyl ester	2.21 ± 0.01	—	—
2-Hexyldecyl propionate	—	—	3.55 ± 0.00
2-Thiopheneacetic acid, oct-3-en-2-yl ester	—	—	1.59 ± 0.00
9-Hexadecenoic acid, 9-hexadecenyl ester	5.37 ± 0.01	3.11 ± 0.01	—
Docosanoic acid nonyl ester	5.59 ± 0.00	—	—

**Table 6:** Comparison of the VOCs of different types of chicken breast.

VOCs	BJY		LH		AA	
	Quantity	Relative Content	Quantity	Relative Content	Quantity	Relative Content
Hydrocarbons	6	34.29%	9	45.68%	19	89.02%
Alcohols	6	31.36%	5	32.32%	1	0.41%
Phenols	1	8.92%	1	13.36%	1	0.06%
Aldehydes	5	12.25%	3	5.53%	2	5.37%

## Data Availability

Data is contained within the article.

## Supplementary Materials

The following are available online at https://www.mdpi.com/article/10.3390/foods11060782/s1, Figure S1: VOCs chromatograms of BJY breast, Figure S2: VOCs chromatograms of LH breast, Figure S3: VOCs chromatograms of AA breast.
