# Supplementary material for: The Use of Electronic Nose in the Quality Evaluation and Adulteration Identification of Beijing-You Chicken
Source: Foods. 2022 Mar 8;11(6):782. doi: 10.3390/foods11060782 (PMC8953052; doi:10.3390/foods11060782)
Supplement: Supplementary file 1 [file foods-11-00782-s001.zip › foods-1536682-supplementary.pdf]

Supplementary Materials

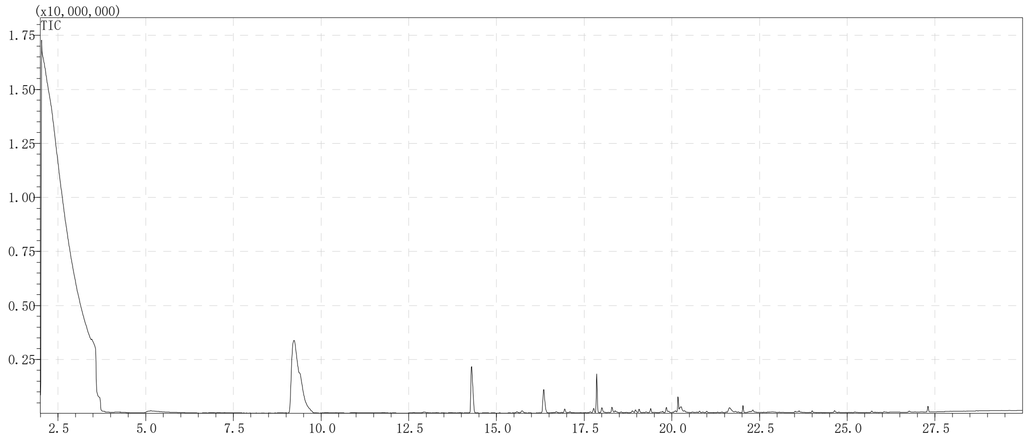

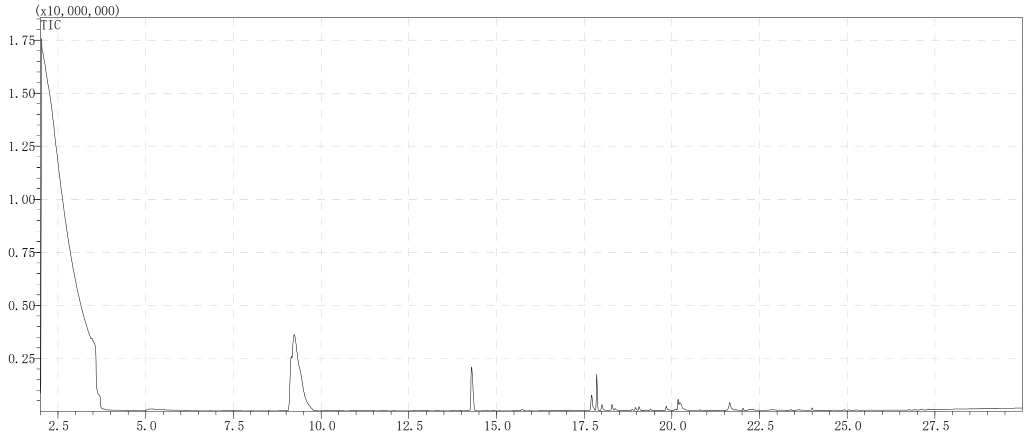

Figure S1. VOCs chromatograms of BJY breast

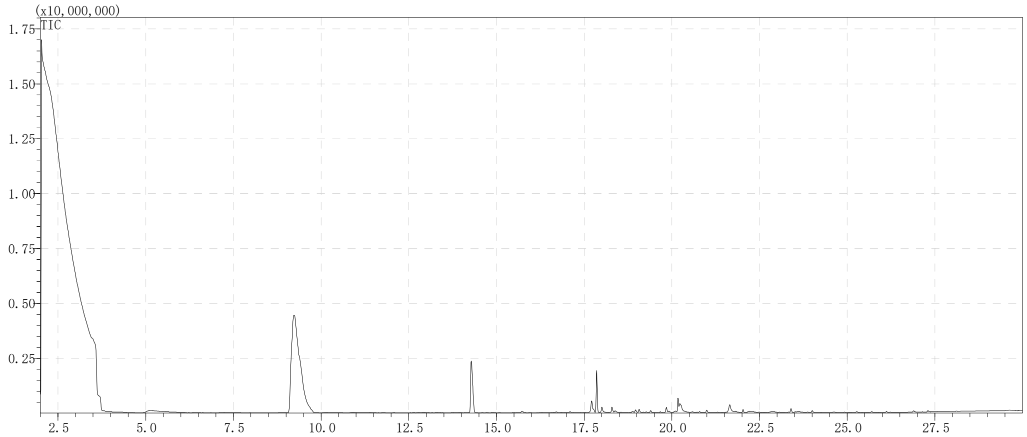

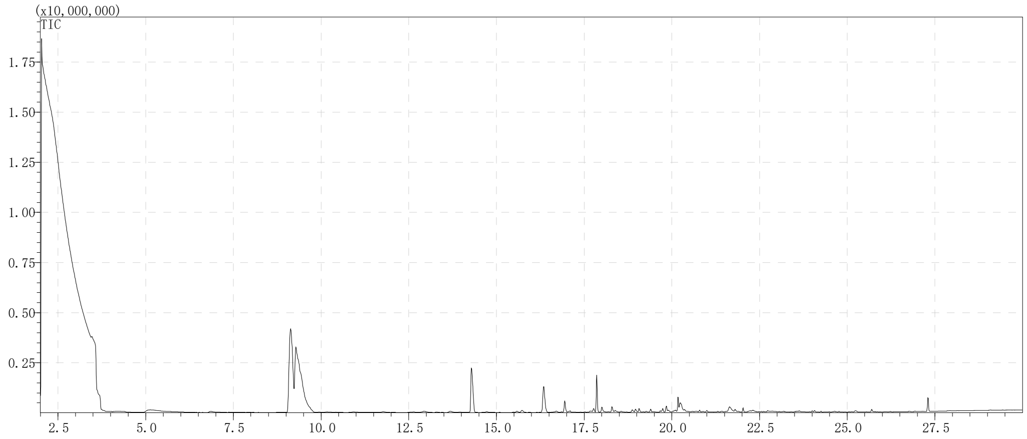

Figure S2. VOCs chromatograms of LH breast

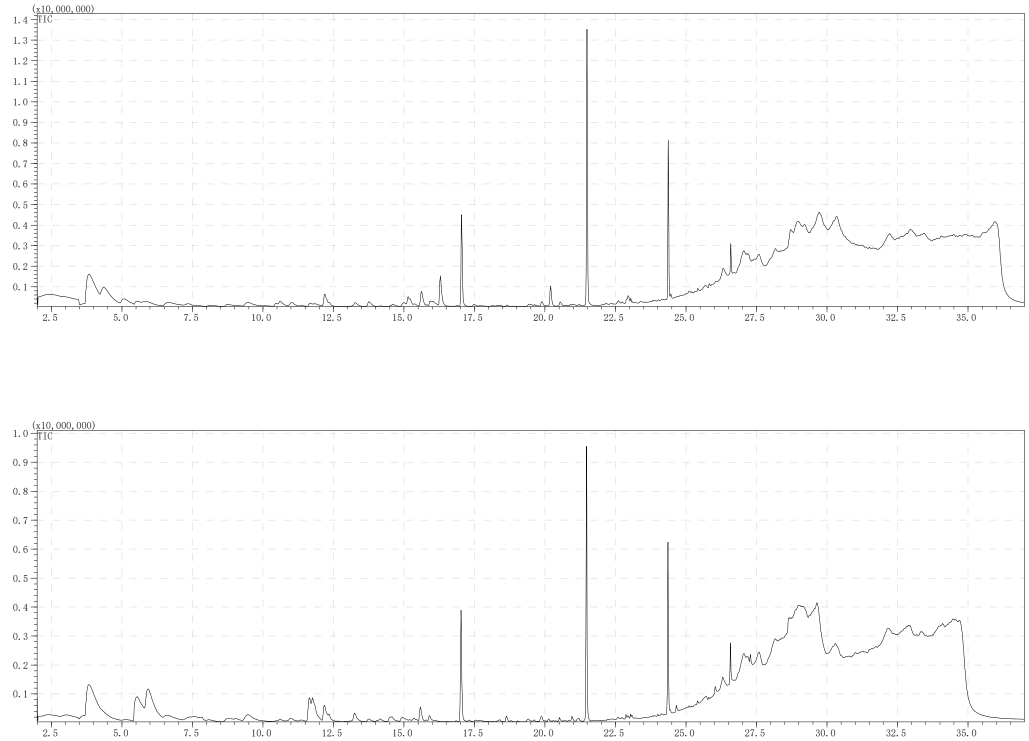

Figure S3. VOCs chromatograms of AA breast
